# Functional and *In Silico* Characterization of ALPL Gene Variants Reveals Genotype–Phenotype Correlations in Italian Hypophosphatasia Patients

**DOI:** 10.3390/cells14221768

**Published:** 2025-11-11

**Authors:** Giulia Casamassima, Anna Maria Grieco, Tommaso Biagini, Giorgia Buono, Luigia Cinque, Flavia Pugliese, Francesco Pio Guerra, Francesco Petrizzelli, Mario Mastroianno, Tommaso Mazza, Marco Castori, Alfredo Scillitani, Vito Guarnieri

**Affiliations:** 1 UOC Genetica Medica, Fondazione IRCCS Casa Sollievo della Sofferenza, San Giovanni Rotondo, 71013 Foggia, Italy; giulia.casamassima96@gmail.com (G.C.); annamar.grieco@gmail.com (A.M.G.); giorgiabuono96@icloud.com (G.B.); luigia.cinque@aslfg.it (L.C.); m.castori@operapadrepio.it (M.C.); 2 Computational Biology and Bioinformatics Unit, Fondazione Policlinico Universitario Agostino Gemelli IRCCS, 00136 Rome, Italy; tommaso.biagini@policlinicogemelli.it (T.B.); tommaso.mazza@policlinicogemelli.it (T.M.); alfredo.scillitani@gmail.com (A.S.); 3 Unit of Endocrinology, Fondazione IRCCS Casa Sollievo della Sofferenza, San Giovanni Rotondo, 71013 Foggia, Italy; flaviapugliese.dott@gmail.com; 4 Laboratory of Oncology, Fondazione IRCCS Casa Sollievo della Sofferenza, San Giovanni Rotondo, 71013 Foggia, Italy; fp.guerra@operapadrepio.it; 5 Laboratory of Bioinformatics, Fondazione IRCCS Casa Sollievo della Sofferenza, San Giovanni Rotondo, 71013 Foggia, Italy; f.petrizzelli@operapadrepio.it; 6 Scientific Direction, Fondazione IRCCS Casa Sollievo della Sofferenza, San Giovanni Rotondo, 71013 Foggia, Italy; m.mastroianno@operapadrepio.it

**Keywords:** enzymatic activity, functional assay, HPP, *in silico* prediction, TNSALP

## Abstract

**Highlights:**

**What are the main findings?**

- First bullet: functional characterization of 21 ALPL genetic variants identified in well clinical ascertained HPP patients that provided novel insights about the protein stability and residual enzymatic activity of the mutants.
- Second bullet: a multifaceted comparison among functional, genotypic, clinical/biochemical and *in silico* data that might represent a higher and more accurate level of a basic genotype-phenotype correlation.

**What are the implications of the main findings?**

- First bullet: a greater comprehension of the presumed effect of an ALPL genetic variant on the whole TNAP protein function.
- Second bullet: the multi-data comparison might represent for the clinician an innovative tool for the follow up and management of a HPP patient.

**Abstract:**

**Background.** Hypophosphatasia (HPP) is a rare genetic disorder caused by impaired tissue non-specific alkaline phosphatase (ALPL/TNSALP) activity that impacts the musculoskeletal and neurological systems. It is extremely variable, with up to six forms of increasing severity. The large phenotypic variability and the still remaining high number of variants of uncertain significance (VUS) in the ALPL gene represent a conundrum for clinicians dealing with people suspected to be suffering from HPP. **Methods.** We applied a multi-faceted bench-based and high-throughput bioinformatics analysis to investigate the effect of 21 ALPL variants (18 deleterious—pathogenic or likely pathogenic—and 3 VUS) on the structure and function of the mutated encoded protein. The results were compared with available clinical and biochemical data. **Results.** Most variants were downregulated or not expressed by Western blot analysis. Impairment of the enzymatic activity was confirmed in vitro for all variants by a specific colorimetric enzymatic assay. *In silico* prediction was in line with functional data and allowed for preliminary categorization of variants based on their impact on both the overall stability of the protein complex and local structural alterations. Coherence among bioinformatics, experimental and clinical data was documented for more than 70% of the variants. **Conclusions.** Functional and *in silico* characterizations of ALPL variants in people with a suspicion of HPP offer integrative strategies to genotyping in assisting clinicians for diagnosis confirmation in doubtful cases.

## 1. Introduction

Hypophosphatasia (HPP, OMIM #146300) is a rare genetic enzymatic disorder of bone and mineralization that may affect the nervous and musculoskeletal system [1]. A decreased level of the tissue non-specific alkaline phosphatase (TNSALP) enzyme causes the disease due to inactivating pathogenic variants of the ALPL (NM_000478) encoding gene [1]. Deficiency in TNSALP phospho-hydrolase activity leads to the accumulation of two main substrates of the enzyme: inorganic pyrophosphate (PPi), a strong inhibitor of bone mineralization, and PLP (pyridoxal 5′ phosphate), the active form of vitamin B6, which, unable to cross the brain barrier, might cause seizures [1].

The multifaceted HPP phenotype presents with different severities and can be classified into up to six classes of increasing severity, ranging from the prenatal, life-threatening, form up to the adult, often misdiagnosed, osteoporosis-like, presentation [1]. The severity depends on the allelic state (i.e., autosomal dominant or recessive inheritance, respectively) and on the possibility that in AD forms, the mutated allele might also impair the enzymatic activity of the healthy allele (dominant negative effect, DNE) [2]. This ultimately leads to a phenotype that is more serious than expected for an AD form. Nevertheless, in sporadic cases or in the absence of sufficient pedigree data, the identification of a single variant in one allele of *ALPL* remains a challenging scenario for clinical interpretation, especially in the case of private or poorly detailed variants.

The latest goals in molecular diagnostics based on the Next-Generation Sequencing (NGS) approach have made it possible to estimate the prevalence of an ALPL variant at about 1/6370 of moderate cases in the European population [3]. A recent initiative is aimed at improving the clinical efficacy of genetic testing in HPP by actively supporting the institution and implementation of a database dedicated to *ALPL* variants [4], accessible at https://alplmutationdatabase.jku.at/ (last release on 5 August 2025). However, this gene still lacks specific adaptations of the American College of Medical Genetics and Genomics/Association of Medical Pathologists (ACMG/AMP) criteria for variant interpretation [5]. Moreover, the mutational repertoire of *ALPL* is wide, with still a high rate of private variants. Therefore, the identification of variants of unknown significance (VUS) in *ALPL* in the absence of any other laboratory findings supporting the diagnosis is a significant risk in the real world. As HPP is a heritable disease that can potentially affect other family members and that features contraindicated and recommended treatments, the generation of laboratory reports with actionable findings is of utmost importance for patients and their families.

In 2023, we described the largest survey of Italian HPP patients, with 23 molecularly confirmed HPP cases, including 8 novel ALPL variants and 1 large genomic deletion [6]. Through the worldwide ACMG interpretation guidelines [5], the 22 different variants were scored as follows: 2 were pathogenic (P), 17 were likely pathogenic (LP) and 3 were variants of uncertain significance (VUS).

In the present work, we offer functional data and more and deeper bioinformatics analyses to verify and support our previous findings. Our investigation strongly supports the need to incorporate functional investigations into the clinical laboratory workout of *ALPL* analysis and to select bioinformatics resources that have been trained on functional data. We also propose some genotype–phenotype correlations in our cohort.

## 2. Materials and Methods

### 2.1. Patients, Clinic and Molecular Analyses

Patients were previously described and clinical details can be accessed through our previous publication [6]. DNA was extracted from blood withdrawal and classic PCR and Sanger or NGS Sequencing were applied to analyze all the subjects (for further technical details, please refer to [6]).

### 2.2. Cell Lines

All functional assays were performed on Human Embryonic Kidney Cells (HEK) 293 (ECACC, Salisbury, UK), as they do not express an endogenous TNSALP [7,8]. Cells were grown at 37 °C, 5% CO_2_, in DMEM + Glutamax (GIBCO, Thermo-Fisher, Waltham, MA, USA) supplemented with 10% FBS (GIBCO) and 1% Penicillin Streptomicin (Sigma-Aldrich, Sant Louis, MO, USA).

### 2.3. Vectors

A basic pcDNA3.1 vector containing the wild type ALPL sequence and encoding for the native protein was used (the vector was kindly gifted by Dr S Graser, Bernhard-Heine-Center for Locomotion Research, Julius-Maximilians-University, Würzburg, Germany). Different pathogenic variants were created by the QuickChange II site-directed mutagenesis kit (Stratagene, Santa Clara, CA, USA), according to the manufacturer’s instructions. The correctness of the mutated vectors was finally verified through colony PCR and Sanger sequencing. As internal controls, 2 well known pathogenic variants, A179T and the E235G were included [9,10].

### 2.4. Western Blot

750 thousand HEK293 cells were seeded in six well plates and, the day after, transfected with 1.5 ug DNA of WT or mutants TNSALP using Lipofectamine 3000 Transfection reagent (ThermoFisher), following the manufacturer’s instructions. Forty-height hours after the transfections, whole crude protein lysate extracts were obtained in RIPA buffer [150 mM NaCl, 50 mM Tris-HCl, 1%, Nonidet P-40, 0.1% sodium dodecyl sulfate (SDS), 0.5% sodium deoxycholate, pH 8.0] supplemented with Complete EDTA, phosphatase and protease inhibitor and PhosStop (both from Roche, Mannheim, Germany, both 1 tablet/10 mL). Proteins were denatured at 70 °C for 10′ and then loaded onto a 10% SDS polyacrylamide gel and electrotransferred (Mixed program for 7′, Transblot, Biorad, Hercules, CA, USA). Then the membranes were blotted overnight at 4 °C with primary monoclonal antibody (1:200, anti-TNAP, sc137213, Santa Cruz Biotechnology, Dallas, TX, USA) in Blocking Buffer solution (BB, Tris-Cl 200 mM, NaCl 1.5 M, pH 7.6, Tween20 1% + 5% skimmed milk) or reference (1:200 in BB, beta-actin, sc-517582, Santa Cruz Biotechnology, Dallas, TX, USA) and, the day after, for 1 h at room temperature with secondary antibody (1:7000 in BB, goat anti-mouse, Biorad, Hercules, CA, USA). Chemiluminescent reaction was obtained with Pierce (ThermoFisher) and signal detection through the Chemidoc System (Biorad).

### 2.5. Degradation of Truncating Mutants

With regard to the truncating variants (S310fs, Y285*, R391fs, and K322fs), we investigated whether they were sensitive to proteasome-mediated degradation, as expected for other mutants [11]. Thus, we repeated the western blotting under the same conditions, but in the presence of the bortezomib, a known strong proteasome inhibitor [12]. Twenty-four hours after the transfection, the drug was added (Selleck, Houston, TX, USA, 50 uM final concentration), protein extraction was made after other 24 h, and lysates were loaded onto a 7.5% SDS PAGE and blotted as described above.

### 2.6. Colorimetric Enzymatic Assay (CEA)

Fifty thousand HEK293 cells were seeded in 96 wells plates and, the day after, transfected in eight replicates with 75 ng of WT or mutants’ vectors. For the F290L/R136H and E191K/K322fs genotypes found in two families with AR-HPP, co-transfections of the WT/variant or variant1/variant2 (35 ng of each vector) were performed. After 48 h, Residual Enzymatic Activity (REA) was measured using the Amplite Colorimetric Alkaline Phosphatase Assay Kit (Yellow Color, AAT Bioquest, Pleasanton, CA, USA) following the manufacturer’s instructions.

### 2.7. Modelling

The atomic coordinates of the TNSALP protein in its dimeric form were obtained using the specialized AlphaFold-multimer model [13]. Structural damage caused by missense mutations was characterized using the Missense3D mutation web tool [14]. It reports all putative changes in structural features introduced by the amino acid substitution, providing an estimate of the structural impact of the variant. Finally, the thermodynamic stability of each mutant structure was investigated using the BuildModel function implemented in the FoldX algorithm [15], which was run with standard parameters.

FoldX computed the total energy of both monomeric and dimeric TNSALP protein, wild-type and mutated, as a proxy for their overall stability and the van der Waals interresidue clashes, as energy penalization factors. Thus, both pdb structures were minimized before assessing their stability, that is, all the side chains were slightly moved in order to reduce the Van der Waals’ clashes. Mutations were classified as destabilizing when ΔΔG exceeded +0.61 kcal/mol for the monomer and +1.2 kcal/mol for the dimer. Conversely, mutations were considered stabilizing when ΔΔG values were below −0.61 kcal/mol (monomer) and −1.2 kcal/mol (dimer) (Table 1).

### 2.8. Statistical Analysis

For western blot, the Image Lab software (version 6.1, Biorad) was used for signal densitometry of 88 and 66 kDa bands of the TNSALP protein. Data were derived from triplicate experiments, were normalized with respect to the WT and the Mann–Whitney U test was performed to statistically compare the values. For CEA assay on eight replicates for each vector, Welch’s *t*-test (two-tailed) was used. For both the tests a significance of 0.05% and confidence level of 95% were considered.

## 3. Results

### 3.1. Overview of the Patients’ Cohort

The cohort under study consists of 23 patients with molecular HPP diagnosis: 17 females/6 males, sex ratio F/M = 2.8, with a median age at last examination of 47.8 years (±22.8 standard deviation) and a median age at diagnosis of 43.9 years (±22.9 standard deviation). For 10 out of 23 cases (43.4%), the segregation analysis was extended to relatives with all instances of inheritance of the variant from an affected individual. For the remaining, it was not possible to recruit other relatives and, thus, the disease origin was not investigated.

Thus, overall, the patients’ cohort included 13 apparently sporadic and 10 familial cases with two (or more) affected individuals. Apart from the patient carrying a large genomic deletion of the exon 2, in 17 other cases, genetic testing identified a single, heterozygous variant. Among them, in 8 cases (47%), segregation of the variant from one (or more) affected individuals was in support of an autosomal dominant inheritance pattern. Among the 17 monoallelic variants, their clinical interpretation after functional investigations was pathogenic in 1, likely pathogenic in 14 and VUS in 2. In 2 cases (8.6%), we found biallelic variants, comprising compound heterozygous genotypes in both the pedigrees. Among the 4 variants identified in biallelic genotypes, their clinical interpretation after functional investigations was pathogenic in 1, likely pathogenic in 2 and VUS in 1.

### 3.2. TNSALP Expression Patterns

It is well known that TNSALP undergoes a trafficking process towards the membrane [9]. Thus, the protein is present, in different forms, in the cell: (i) a basic cytoplasmic structure of 58 kDa; (ii) a glycosylated form of 66 kDa that emerges from the post-translational modification of the Endoplasmic Reticulum (ER); (iii) a further mannosidated form of 88 kDa, as a result of the modifications into the Golgi [9]. Moreover, it is known that into the ER, the TNSALP dimerizes and, after the step into the Golgi, the final homodimer reaches and anchors to the membrane through the GPI [9,16].

**Figure 1:**
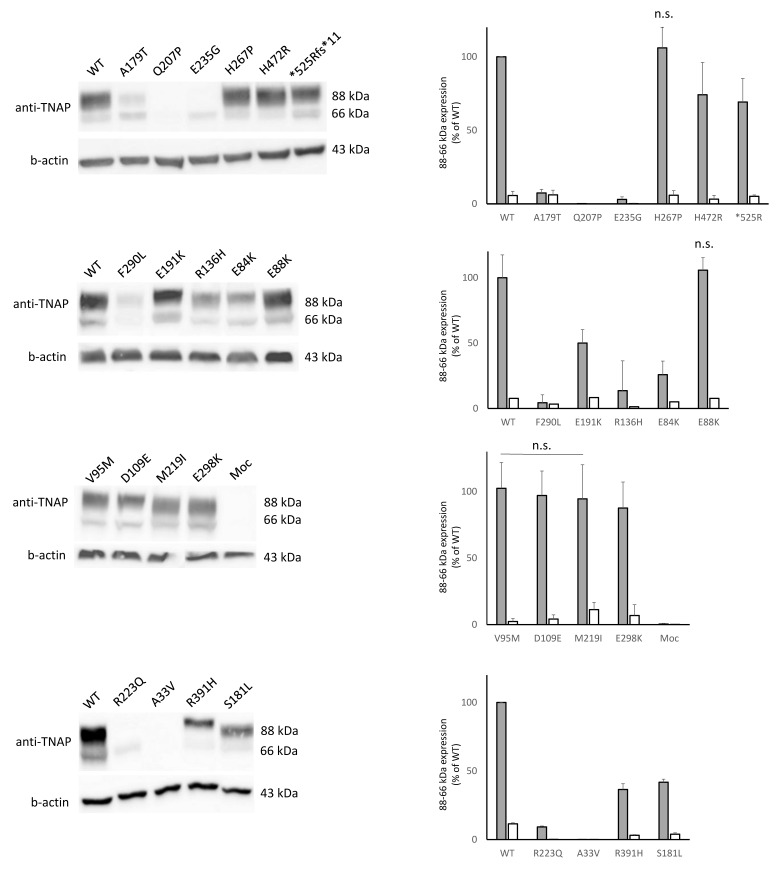
Western blot showing the expression of 66 and 88 kDa bands (on the **left**). Comparison, as relative expression (% of 88 or 66 kDa WT band) of mutants and quantification are plotted (on the **right**). Unless specified, all the comparisons are statistically significant; n.s. = not significant (for one of the two bands or for both).

In Figure 1, both the bands, 66 and 88 kDa, were shown. Looking at the WT, the lower band (at 66 kDa) is less expressed with respect to the higher one (88 kDa), indicating that the protein underwent proper trafficking from the ER towards the Golgi [9]. Interestingly, we observed a differential expression of other mutants compared to the WT; 7 mutants (E88K, V95M, D109E, H267P, E298K, H472R, *525Rfs) showed a comparable expression for both the bands with the WT or even higher expression (H267P, H472R). Other 7 mutants (E84K, R136H, S181L, E191K, M219I, F290L and R391H) showed decreased intensity of both bands. Seven mutants (A33V, Q207P, R223Q, S310fs, K322fs, Y285*, and R391fs) were barely expressed, less than the 10% compared to the WT for both the bands. For the 2 internal negative controls (A179T, E235G) while the 66 kDa showed an intensity similar to that of the WT, the higher 88 kDa appeared faint, thus suggesting an impaired translocation towards the Golgi, with likely ER accumulation, in line with what was previously reported at least for the A179T [9]. According to the TNSALP expression pattern analysis, a loss-of-function effect, either in the form of hypomorphic or *null* alleles, might be considered for variants with decreased or absent intensity of both bands.

### 3.3. Test with Bortezomib

**Figure 2:**
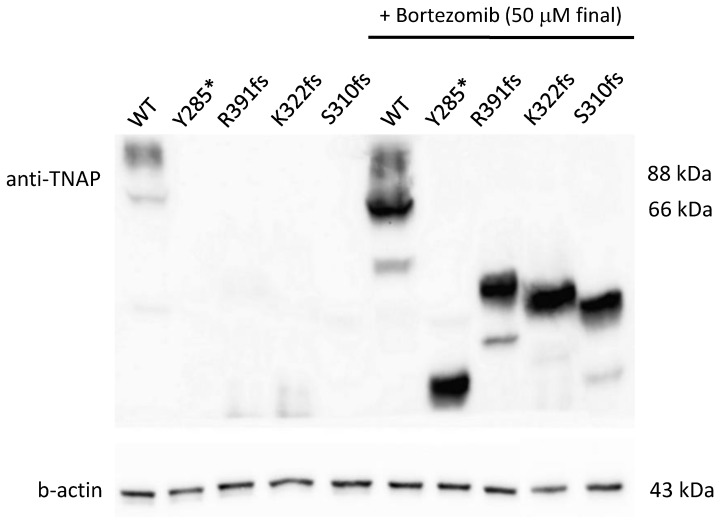
Western blot on truncating variants treated or not with bortezomib. In not treated cells, the western blot shows a pretty negligible expression of 66 and 88 kDa bands for all the mutants (on the **left**); after the treatment, bands are visible with a corresponding size to the residual length of the mutated protein (on the **right**).

Proteasome inhibition with bortezomib at 50 uM (final concentration) for 24 h confirmed that all the 4 truncating mutants (Y285*, S310fs, K322fs and R391fs) were subjected to a proteasome degradation, since, under treatment, all the mutants were visible with bands lower than the regular ones depending on the residual length of the truncated protein (Figure 2).

### 3.4. Enzymatic Activity

**Figure 3:**
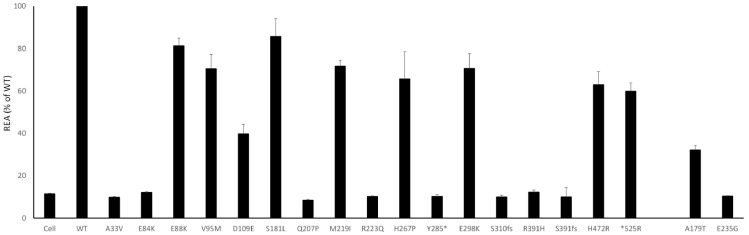
Residual Enzymatic Activity (REA) are plotted (% of WT) for each mutant; standard deviation was calculated and plotted as well. All the values were significant (*p* < 0.005). As expected, the REA for truncating and stopgain variants is in the range of the background noise of the not treated cells.

The assay showed an impairment of the enzymatic activity for all mutants. Concerning missense variants, the decrease of enzymatic activity ranged from 20% for E88K to almost total loss of activity for the E84K. In line with their presumed total loss of function, mutants Y285*, S310fs, K322fs, and R391fs showed a REA similar to the background noise of untreated cells (Figure 3), suggesting that these mutants could be considered as additional internal controls, confirming the reliability of the assay.

### 3.5. Families with Compound Heterozygotes

**Figure 4:**
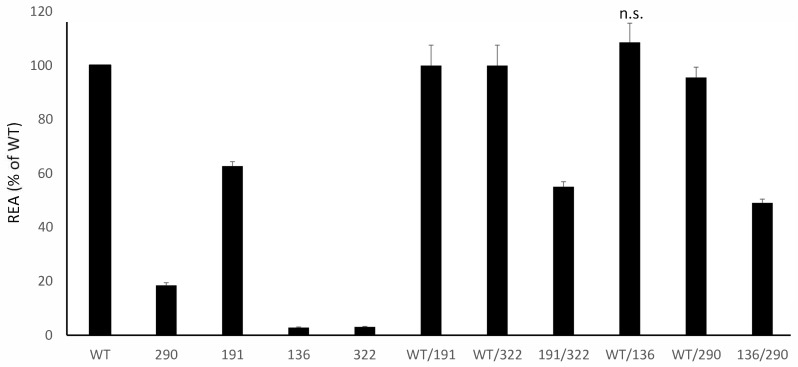
Residual Enzymatic Activity REA of mutants identified in compound heterozygotes patients, tested alone (on the **left**) and in combination (on the **right**) and plotted (% of WT). Please see the text for further comments; unless specified, all the comparisons are statistically significant; n.s. = not significant.

Two probands inherited the disease in an autosomal recessive manner, with compound heterozygotes for two different variants (i.e., R136H on one allele and F290L in the other; E191K on one allele and K322fs in the other). In both cases, the disease had an onset in childhood and the cumulative CEA of the two deleterious variants was in line with an autosomal recessive inheritance with minimal or absent clinical manifestations in the parents (Figure 4) [17]. The assay was also able to confirm that the E191K variant has no dominant negative effect on the WT allele, as previously defined [18].

### 3.6. In Silico Pathogenicity Assessment

The combined *in silico* analysis using Missense3D and FoldX allowed the missense variants to be divided into two groups. The first group (S181L, H267P, G491R, D109E, R223Q, G426D, and Q207P) was classified by Missense3D as damaging, as these substitutions disrupted key interactions in buried regions or perturbed secondary structure elements. Notably, the D109E variant, located in proximity to the active site valley, also induced an alteration in cavity volume. For all of these variants, FoldX also highlighted significant changes in protein stability, showing a clear concordance between the two structural predictors and experimental data, such as expression levels or reduced residual enzymatic activity.

The second group includes the variants A33V, Y28D, R272H, R136H, F290L, H472R, A179T, P174L, P307L, M219I, R391H, and E84K for which, Missense3D did not show any significant structural changes. Nevertheless, FoldX analysis revealed a relevant effect on protein stability in both monomeric and dimeric forms. In all cases, the |ΔΔG| values exceeded the absolute classification thresholds (0.61 kcal/mol for the monomer and 1.2 kcal/mol for the dimer), indicating substantial changes, either destabilization (positive ΔΔG) or abnormal stabilization (negative ΔΔG). Such deviations, regardless of sign, are consistent with the experimentally observed reduction in protein expression levels, suggesting that both excessive destabilization and overstabilization may impair correct folding and/or promote aberrant degradation of the mutant proteins. (see “Missense3D Prediction” and “Missense3D Structural Damage” columns in Table 1).

### 3.7. In Silico Data Propose a Variant Categorization

Based on structural evaluation, *in silico* analysis provided a possible categorization of the variants into three main groups: structurally perturbed variants, stability-impacting variants and active-site/interface-proximal variants.

Structurally perturbed variants (SP): The first group (D109E, S181L, Q207P, H267P, and R223Q) showed a clear concordance between the two structural predictors, as well as with experimental data, that is, expression values or reduced residual enzymatic activity.

The second group of stability-impacting variants (SI) (A33V, E84K, R136H, A179T, M219I, F290L, R391H, and H472R) affected the stability of the protein, both in monomeric and dimeric forms, in concordance with the lower expression of the protein.

Finally, active-site/interface-proximal variants (A-SI) (E88K, V95M, E191K, E235G, and E298K) showed minimal or zero perturbations in stability, mainly related to the dimeric form. These results are consistent with their localization, as energetic calculations and static structure evaluations cannot accurately capture the impact of variants on a functional region, requiring further studies that include protein dynamics and conformational flexibility.

**Figure 5:**
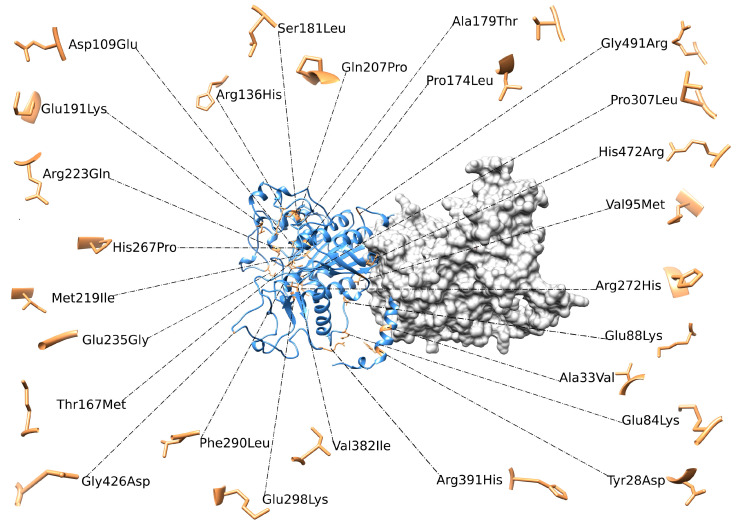
3D structure of ALPL dimer obtained through molecular modeling. Mutations were mapped on the wild-type structure and highlighted in light brown.

All these variants are represented in Figure 5. The p.(*525Argextfs*11) variant is a stop-loss frameshift mutation that results in a C-terminal extension of 11 amino acids. Because the resulting structure differs substantially from that of the wild-type protein and given that tools such as Missense3D and FoldX provide reliable results only for small structural perturbations typically induced by missense variants, this variant cannot be classified into the previously described groups and, therefore, was not analyzed. In fact, its characterization requires a dedicated analytical approach aimed at modeling the 11 amino acids extension and predicting the correct folding and overall protein stability.

### 3.8. Genotype-Phenotype Correlations in This Cohort

About the two compound heterozygous forms, it is likely the both the variants contributed separately to the whole phenotype of the affected patients. As far as the other 19 identified variants, including 17 candidate variants and 1 known variant with a previously assigned pathogenicity according to ACMG criteria (R391fs), some considerations on their deleterious effect could be put forward by intersecting clinical, family and functional data (Table 2). More specifically, 11 variants, also comprising the novel changes E84K, Y285*, F290L and S310Pfs*, present functional data compatible with haploinsufficient or severely hypomorphic alleles, with a CEA value ranging from 0% to 25%. For these variants both monoallelic (9 variants, A33V, E84K, Q207P, R223Q, Y286*, S310fs, R391H and R391fs) and biallelic (3 variants, R136H, F290L and K322fs) inheritance has been documented or inferred by combining clinical data with the resulting genotype. Among patients presenting these variants, childhood onset was registered in the two biallelic cases as well as in a 6-year-old subject carrying the heterozygous R391H variants. In all other cases, disease onset occurred in young-adult to adult age (17 to 38 years). Considering the 9 variants with CEA values ranging from 40% to 80%, we had the E88K, V95M, D109E, E191K, M219I, H267P, E298K, H472R and *525R. In all of them, the age at onset ranged from 30 to 50 years with two exceptions. The V95M variant was observed in a 12-year-old individual and was associated with a CEA value of 40%. The other exception is the E88K variant observed in two families; in one, the disease onset was at 50 years while in the other the disease presented more seriously at 10 years with nephrolithiasis/calcinosis. Therefore, by excluding the V95M variant which showed CEA values still compatible with a significantly hypomorphic allele, the remaining had a residual enzymatic activity ranging from 60% to 80% and predominantly associated with a more advanced age at onset at least in the majority of cases. Early onset, motor difficulties, convulsions, *genua valga* and failure to thrive, which may be considered proxies for an early-onset and likely more severe phenotype characterized by developmental attributes and higher risk of long bone deformities, always associated with CEA ranging from 0% to 25%. On the other hand, phenotypes with a later onset characterized by a variable association of musculoskeletal pain, fractures/reduced bone mass and/or edentulia, are associated with CEA ranging from 40 to 70–80%. From a graphical point of view (Table 2), we attributed a color gradient towards the red for:

- severity of the symptoms: “early onset”, “fractures (vertebral or multiple)”, “neurodevelopmental disorders” and “failure to thrive” are considered more severe symptoms (red) than “late onset”, “low bone mass”, “osteoporosis”, “edentulia” and “others” (orange). “Lack of symptoms” are in pale blue;
- the increasing impairment of the functional data (expression at the WB, REA or ΔG) of the modelled mutated protein; normal expression/REA > 80%/ΔGdim < 0 in green; down expression/REA > 40% but < 80%/ΔGdim < 6 in orange; loss of expression/CEA < 40%/ΔG > 6 in red;
- the different HPP classes from the life-threatening perinatal (red), through the early onset (childhood and infantile) (orange) forms up to the mild adult presentations (light green).

Combining all these results, for at least 12 out of 17 variants (70.5%; A33V, D109E, Q207P, M219I, H267P, Y285*, E298K, S310Pfs*28, R391H, R391P*fs14, H472R and *525Rfs*11) a possible trend of genotype/functional/phenotype correlation was inferred. Moreover, consistency was also found for variants inherited by recessive fashion in the Family I (R136H/F290L).

### 3.9. Comparison with Literature Data

A search into public database pointed out that, of our set of 21 variants, 8 (38%, V95M, R136H, S181L, E191K, M219I, R223Q, R391H and H472R) have been reported in HPP AD forms (Table 3). Taking into account the known incomplete penetrance and the variable expressivity, it is not surprising that only 4 variants caused the same HPP phenotypic class (S181L, 1 childhood; M219I, 1 adult; R223Q, 2 adult; R391H, 1 childhood) while the remaining 13 variants (62%), had been found either in HPP AD and AR cases (in homozygosity or compound heterozygosity), with variable clinical presentation.

Finally, a comparison of our REAs with the values recorded into the database showed concordance for 6 variants out of the 13 tested (46%, Table 1). Whether this discrepancy was due to the difference in the technical detection method (testing the crude lysates protein rather than using living cells) or to other variables, is not known and it is out of the aims of this current paper.

## 4. Discussion

In this study, we applied a parallel approach consisting of bench-based functional assays on HEK293 cells and high-throughput bioinformatics analysis with the aim to provide a comprehensive characterization of 23 *ALPL* deleterious or potentially deleterious variants identified in a large survey of Italian HPP patients [6]. Seven out of 21 variants (33.3%, E84K, H267P, Y285*, F290L, S310Pfs*28, R391Pfs*14 and *525Rfs*11) were not reported in the official database (https://alplmutationdatabase.jku.at, last release: 5 August 2025). In addition to these 7 novel variants, in this work, we added expression study values and REA characterization for 12 and 6 previously published variants, respectively.

We implemented different molecular assays to: (i) investigate the degree of maturation of the mutated TNSALP; (ii) determine the corresponding REAs; (iii) explore whether some truncating mutants were catabolized via a proteasome-mediated pathway, as previously reported for other TNSALP mutants [11]. Then, the effect of the variant on the 3D structure was deduced through different modeling approaches, and in the same way, quantified by a structural destabilization index such as the ΔG value. Finally, all the data were compared with phenotypes and an overall correlation among all the results was attempted.

### 4.1. Consistency of Functional and In-Silico Results

At the WB, mutant proteins showed a differential pattern of expression, ranging from the total loss (for some missense and all the truncating causing variants) to down and normal expression (for other ones). Importantly, the different REAs were in line with the WB results and both observations were corroborated by *in silico* predictions. For the totally-not expressed frameshift mutants (Y285*, S310Pfs*28, K322Rfs*44 and R391Pfs*14), we confirmed that they are degraded through the proteasome pathway. However, further studies on the mechanistic effects of A33V and F290L on the stability of the protein and/or of the mRNA, are required. According to this work, the three variants H267P, F290L and *525Rfs*11, that were previously classified as VUS [6], were reclassified all as likely pathogenic by adding the PS3_Supp criterion (“well-established functional studies show damaging effect on the gene or gene product”) [5].

### 4.2. Crossing Functional/In Silico Data with Phenotypes

The consistency of bench/*in silico* results suggested a possible correlation between the two sets of data. Instead, in more than the 70% of cases (12 out of 17 variants, all found as monoallelic state, Table 3) the level of protein function, in terms of expression, enzymatic activity and predicted de/over-stabilized structure was in line with the clinical portrait of the corresponding patients. This correlation involved, among others, three out of four truncating variants (R391Pfs*14, S310Pfs*28 and Y285*), three missense variants that have been demonstrated to localize at the Ca^2+^ binding domain (M219I, H267P and E298K) [8,19] and two variants affecting the homodimer interface (R391H and H472R) [20]. Concerning the two AR cases, in both at least one identified variant (i.e., R136H in one case and R391H in the other) has been previously described in heterozygous affected individuals (Table 3). In our cases, the presence of a variant in the second allele may contribute to an earlier-onset. On the other hand, the absence of symptoms in the carrier parents in our families suggest that these two variants may act as hypomorphic alleles leading to milder and incompletely penetrant phenotypes at the monoallelic state and associate with more severe phenotypes only in combination with a second deleterious allele.

As expected, a more adherent correlation exists for cases with early onset and carrying definitive pathogenic (i.e., truncating/frameshift) or for AR cases with biallelic variants. Unexpectedly, adult forms share the same clinical spectrum consisting of low bone mass, osteoporosis, musculoskeletal pain and other non-classic manifestations, such as nephrolithiasis.

### 4.3. Data Comparison with Literature

Sixteen identified variants were already reported in the *ALPL* database. Seven of them were associated with both autosomal dominant and recessive inheritance patterns with a variable age at onset, which appeared grossly unrelated to the allelic state. Four variants were annotated in the database at the monoallelic state only, while the remaining five occurred in instances of autosomal recessive inheritance only. In our cases with monoallelic involvement of *ALPL*, we excluded a copy number variation in the second allele by MLPA and review of the sequencing data. Scrutiny of the *ALPL* database and the two biallelic cases described here document that the inheritance pattern of HPP is largely unpredictable by considering the genotype only. While several variants seem sufficiently deleterious by generating a full-blown phenotype at the monoallelic state, many others occur at the monoallelic and biallelic state without a strong correlation with the presenting phenotype. Therefore, family segregation studies and second-tier investigations aimed at excluding a second hit which run undetected at standard diagnostics are crucial for inheritance pattern discrimination. In case of monoallelic genotypes and non-contributory family study, in vitro investigations with or without preceding *in silico* analysis might contribute to exploring a coherence between phenotype and allelic state. Our work, while supporting the existence of some linear relationships among *in silico* prediction, functional data, genotype and phenotype, did not identified a rule. Therefore, other factors likely exist that contribute to such an intrafamilial and interfamilial variability of HPP and maintain its diagnosis a complex procedure in which genetic testing is not always clarifying.

### 4.4. Reliability and Limits of the REA Values

As far as the determination of the REA, at the variance with what done with other assays based on total protein lysates [2], a commercial kit that tests living cells was implemented. One limitation of this kit, which is common to other commercial assays, is the use of a synthetic substrate (pNPP). On the other hand, the colorimetric test used in this current work allowed determining only the enzymatic activity of the fraction physiologically active, actually localized at the plasmamembrane. Thus, we believe that our test provided a reliable picture of the REA, so that it was able to infer the genotype-phenotype correlation for the two AR HPP families.

## 5. Conclusions

Here we expanded the molecular repertoire of ALPL and confirmed that a combination of functional assays and high-throughput bioinformatics may significantly contribute to determine the stability, structure/function of the corresponding mutated protein. The preliminary data on phenotype/genotype/*in silico*/functional intersections might pave the way to a multidimensional approach to laboratory testing in HPP aimed at improving the actionability of genetic testing especially in case of VUS.

## Figures and Tables

**Table 1:** Functional, biochemical and modeling data of the TNSALP mutants; A-SI, active-site/interface. CEA, Colorimetric Enzymatic Assay; Concord, concordance; DCD, developmental coordination disorder; NA, not available; SI, stability-impacting variants; SP, structurally perturbated variants; A-SIP, active-site/interface-proximal variants; WB, Western Blot; ↓: level of decreased expression; #: refers to the patients in [6]; § and §§: variant absent and data from ALPL database [https://alplmutationdatabase.jku.at/ (last release on 5 August 2025)], respectively; Significant results at *in silico* analyses were in *italics*. In bold the variants for whom a genotype/functional/phenotype correlation was inferred.

*ALPL* Variant	Allelic Status Mono/Bi	Serum ALP (IU/l)	Onset (Patient = Years)	Musculoskeletal Pain (+/- Muscle Fatigue)	Reduced Bone Mass	Long Bones Fractures (2 or More)	Vertebral Fractures (1 or More)	Premature Loss of Teeth	Nephro-litiasis	Motor Delay DCD	Failure to Thrive	Genua Valga	Others	WB (66/88 kDa)	CEA	Missense 3D Prediction	FoldX Monomer	FoldX Dimer	Group	Database REA Values (Concor) §§
A33V	Mono	38 (↓)	55	-	+	+	+	-	+	-	-	-	-	↓↓↓ ↓↓↓	0%	Neutral	*8.63*	*14.19*	SI	12% (Yes)
E84K §	Mono	19 (↓)	NA	-	+	-	-	-	-	-	-	-	-	↓ ↓	0%	Neutral	*2.97*	*5.25*	SI	NA
E88K	Mono	#7 = 25 (↓) #23 = 63	#7 = 50 #23 = 10	-	+	+	+	-	+ (#23)	-	-	-	Deafness (#23)	Normal Normal	80%	Neutral	−0.85	*−1.86*	A-SIP	NA
V95M	Mono	36 (↓)	12	+	+	+	-	-	-	-	-	-	-	Normal Normal	70%	Neutral	*0.96*	*−1.5*	A-SIP	56% (No)
**D109E**	**Mono**	**#6 = 21 (↓)** **#9 = 21 (↓)**	**#6 = 49** **#9 = 49**	**+ (#9)**	**+**	**+ (#6)**	**-**	**-**	**+ (#6)**	**-**	**-**	**-**	**Hypercalciuria (#6)**	**Normal** **Normal**	**40%**	***Damaging***	***2.12***	***4.87***	**SI**	**NA**
**R136H**	**Bi** **(+F290L)**	**18 (↓)**	**10**	**+**	**+**	**-**	**-**	**-**	**-**	**-**	**-**	**-**	**-**	**↓** **↓**	**0%**	**Neutral**	***7.15***	***7.55***	**SP**	**21.2–33.4% (No)**
**Q207P**	**Mono**	**147**	**0 (birth)**	**+**	**+**	**+**	**-**	**-**	**-**	**-**	**-**	**-**	**-**	**↓↓↓** **↓↓↓**	**0%**	***Damaging***	***5.31***	***8.74***	***SI***	**NA**
S181L	Mono	129	0 (birth)	-	-	-	-	-	-	-	-	-	Pectus excavatum, scoliosis	↓ ↓	85%	*Damaging*	*2.79*	*2.5*	SI	1.3–55.3% (No)
E191K	Bi (+K322fs)	20 (↓)	2	+	-	-	+	-	-	-	+	+	-	Normal Normal	62%	Neutral	−0.61	−1.2	A-SIP	21.4–88% (Yes)
**M219I**	**Mono**	**19 (↓)**	**45**	**+**	**+**	**+**	**-**	**-**	**-**	**-**	**-**	**-**	**-**	**Normal** **Normal**	**70%**	**Neutral**	***2.5***	***4.64***	***SP***	**30.1% (No)**
R223Q	Mono	#1 = 21 (↓) #16 = 4 (↓)	#1 = 20 #16 = 51	+ (#1)	+ (#16)	-	-	-	+ (#16)	-	-	-	-	↓↓↓ ↓↓↓	0%	*Damaging*	*5.52*	*12.15*	*SI*	2.9–5% (Yes)
**H267P §**	**Mono**	**33 (↓)**	**56**	**+**	**+**	**-**	**-**	**+**	**-**	**-**	**-**	**-**	**-**	**Normal** **Normal**	**60%**	***Damaging***	***4.45***	***9.1***	***SI***	**NA**
Y285* §	**Mono**	**23 (↓)**	**17**	**-**	**-**	**-**	**-**	**+**	**-**	**+**	**-**	**-**	**-**	**↓↓↓** **↓↓↓**	**0%**	**NA**	**NA**	**NA**	**NA**	**NA**
**F290L §**	**Bi** **(+R136H)**	**18 (↓)**	**10**	**+**	**+**	**-**	**-**	**-**	**-**	**-**	**-**	**-**	**-**	**↓** **↓**	**10%**	**Neutral**	***7.15***	***7.55***	**SP**	**NA**
**E298K**	**Mono**	**25 (↓)**	**30**	**+**	**-**	**-**	**-**	**-**	**-**	**-**	**-**	**-**	**-**	**Normal** **Normal**	**60%**	**Neutral**	***−0.95***	**−0.3**	**A-SIP**	**23.7% (No)**
**S310Pfs §**	**Mono**	**24 (↓)**	**14**	**+**	**-**	**-**	**-**	**-**	**-**	**-**	**-**	**-**	**-**	**↓↓↓** **↓↓↓**	**0%**	**NA**	**NA**	**NA**	**NA**	**NA**
**K322fs**	**Bi** **(+E191K)**	**20 (↓)**	**15**	**-**	**-**	**-**	**-**	**+**	**-**	**-**	**+**	**+**	**-**	**↓↓↓** **↓↓↓**	**0%**	**NA**	**NA**	**NA**	**NA**	**NA**
R391H	Mono	81 (↓)	6	-	-	-	-	+	-	+	-	-	Convulsions	↓ ↓	5%	Neutral	*1.5*	*4.07*	SP	2.1–3.7% (Yes)
**R391fs §**	**Mono**	**27 (↓)**	**38**	**+**	**+**	**+**	**+**	**+**	**-**	**-**	**-**	**-**	**-**	**↓↓↓** **↓↓↓**	**0%**	**NA**	**NA**	**NA**	**NA**	**2.1% (Yes)**
**H472R**	**Mono**	**8 (↓)**	**37**	**+**	**+**	**-**	**-**	**-**	**+**	**-**	**-**	**-**	**-**	**Normal** **Normal**	**65%**	**Neutral**	***0.64***	***4.33***	**SP**	**38% (No)**
*525R §	**Mono**	**41 (↓)**	**NA**	**+**	**-**	**-**	**-**	**-**	**-**	**-**	**-**	**-**	**-**	**Normal** **Normal**	**60%**	**NA**	**NA**	**NA**	**NA**	**NA**
A179T (control)	Mono	NA	NA	NA	NA	NA	NA	NA	NA	NA			NA	Normal ↓↓↓	25%	NA	NA	NA	SP	64 (No)
**E235G** **(control)**	**Mono**	**NA**	**NA**	**NA**	**NA**	**NA**	**NA**	**NA**	**NA**	**NA**			**NA**	**Normal** **↓↓↓**	**0%**	**NA**	**NA**	**NA**	**A-SIP**	**3.3–3.6% (Yes)**

**Table 2:** Graphical description of the trend of genotype/functional/in-silico/phenotype correlations. Color gradient towards the red indicates an increase severity of the symptoms or of the protein function impairment (expression, enzymatic activity and modelling; i.e., WB in red = total loss of protein expression vs. green = normal expression). In pale blue the asymptomatic cases. Accordingly, HPP classes are visualized with a color gradient depending on the severity: red for early onset and life threatening “perinatal” forms, orange for “childhood” and “infantile” and light green for, less severe, “adult” forms. Finally, a gradient from yellow (low/medium-low) through light green (medium/high) to green (high) is used to make evidence of the level of correlation between the severity/paucity of the symptoms, the protein function and the HPP class; * both reported in two HPP AR cases in compound heterozygosity; NA, not applicable since no clinical data are available (please see the text paragraph 3.7 for details); ** for truncating variants we considered the final effect of a dramatic destabilization, rather than the determination of ΔG that is not theoretically applicable.

Variants	No Symptoms	Early Onset HPP	Late Onset HPP	Edentulia	Reduced Bone Mass	Vertebral Fractures	Multiple Fractures	Failure to Thrive	Neurodevelopmental Disorders	Others	Western Blot	CEA	Bioinfo	HPP Class	Geno/Funct/Pheno Correlation
A179T														perinatal *	no corr
E235G														adult *	NA
															
E191K K322Rfs*44														infantile	low
													**		
E88K														childhood	low
V95M														childhood	low
R223Q														adult	low
E84K														adult	low
S181L														infantile	medium/low
Q207P														perinatal	high
R136H F290L														childhood	high

Y285*													**	childhood	high
S310Pfs*28													**	childhood	high
R391H														childhood	high
A33V														adult	medium/high
R391Pfs*14													**	adult	medium/high
H267P														adult	high
D109E														adult	high
M219I														adult	high
*525Rfs*11														adult	high
E298K														adult	high
H472R														adult	high

**Table 3:** Number of findings and HHP phenotypic class for the variants reported in this current paper compared with the corresponding data in the public database; AD/AR, autosomal dominant or recessive; * parents of the Family II [17]; †, mutants used as internal controls.

	Database		Present Work	
	AD	AR	AD	AR
**Variants**	(number of findings, HPP class)			
A33V	/	2 perinatal, 6 infantile, 2 childhood, 1 adult	1 adult	/
E88K	/	1 infantile	1 childhood	/
V95M	1 adult	/	1 childhood	/
D109E	/	1 adult	2 adult	/
R136H	1 adult, 1 odonto	2 perinatal, 2 infantile, 6 childhood, 3 odontoHPP	/	1 childhood
S181L	1 infantile, 1 childhood, 1 adult	2 perinatal, 5 infantile, 1 childhood, 1 adult, 2 odonto	1 infantile	/
E191K	1 childhood, 2 adult, 1 odonto, 2 asymptomatic *	1 prenatal benign, 12 infantile, 14 childhood, 4 adult, 1 odonto	/	1 infantile
Q207P	/	1P	1 perinatal	/
M219I	1 adult	/	1 adult	/
R223Q	1 perinatal, 1 adult	1 infantile, 1 odonto	2 adult	/
E298K	/	1 perinatal, 1 infantile, 1 adult	1 adult	/
K322R*fs44	1 asymptomatic *	1 perinatal, 1 childhood	/	1 infantile
R391H	1 childhood, 1 adult	/	1 childhood	/
H472R	1 childhood	1 childhood	1 adult	/
E84K	Novel		1 adult	/
H267P	Novel		1 adult	/
Y285*	Novel		1 childhood	/
F290L	Novel		/	1 childhood
S310P*fs28	Novel		1 childhood	/
R391P*fs14	Novel		1 adult	/
*525R*fs11	Novel		1 adult	/
A179T ^†^	1 asymptomatic	1 perinatal	/	
E235G ^†^	1 adult	/	/	

## Data Availability

The original data presented in the study are openly available at doi: 10.3389/fendo.2023.1205977.
